# Supplementary material for: Mechanisms governing the pioneering and redistribution capabilities of the non-classical pioneer PU.1
Source: Nat Commun. 2020 Jan 21;11:402. doi: 10.1038/s41467-019-13960-2 (PMC6972792; doi:10.1038/s41467-019-13960-2)
Supplement: Supplementary file 7 — Source data [file 41467_2019_13960_MOESM7_ESM.zip › Source_Data/FigureS2/FigureS2B_MotifScanOutput/KmeansCluster3/homerResults/motif27.similar.html]

motif27

## Information for motif27

A
C
G
T
A
G
T
C
A
G
T
C
A
G
C
T
C
T
A
G
C
G
T
A
A
C
T
G
T
C
A
G
A
G
T
C
A
G
T
C
C
G
A
T
A
G
T
C
G
A
T
C
  
Reverse Opposite:  

C
T
A
G
C
T
A
G
C
G
T
A
T
C
A
G
A
C
T
G
A
G
T
C
G
T
A
C
A
C
G
T
A
G
T
C
C
T
G
A
C
T
A
G
A
C
T
G
C
G
T
A
  

|  |  |
| --- | --- |
| p-value: | 1e-35 |
| log p-value: | -8.106e+01 |
| Information Content per bp: | 1.923 |
| Number of Target Sequences with motif | 83.0 |
| Percentage of Target Sequences with motif | 1.74% |
| Number of Background Sequences with motif | 94.2 |
| Percentage of Background Sequences with motif | 0.22% |
| Average Position of motif in Targets | 93.6 +/- 78.7bp |
| Average Position of motif in Background | 104.5 +/- 70.4bp |
| Strand Bias (log2 ratio + to - strand density) | -0.6 |
| Multiplicity (# of sites on avg that occur together) | 1.00 |
| Motif File: | file (matrix) reverse opposite |

### Similar de novo motifs found

|  |  |  |  |  |  |  |  |
| --- | --- | --- | --- | --- | --- | --- | --- |
| Rank | Match Score | Redundant Motif | P-value | log P-value | % of Targets | % of Background | Motif file |
| 1 | 0.960 | A C G T A T G C A T G C A C G T T C A G C G T A A C T G C T A G G T A C A G T C G A C T G A T C | 1e-34 | -80.340887 | 2.10% | 0.35% | motif file (matrix) |
| 2 | 0.963 | A C G T A G T C A G T C A G C T C T A G C G T A A C T G T A C G A G T C G A T C C G A T A G T C A G T C A G T C | 1e-34 | -79.091323 | 1.66% | 0.20% | motif file (matrix) |
| 3 | 0.918 | A C G T A T G C T A G C A G C T C T A G G T C A A C T G C T A G A G T C G A T C A C G T | 1e-33 | -76.474156 | 2.14% | 0.40% | motif file (matrix) |
